# NURSING HOME WORKFORCE CHALLENGES: INCREASED NURSING STAFF LABOR COSTS AND USE OF AGENCY STAFF FROM 2017 TO 2021

**DOI:** 10.1093/geroni/igad104.0122

**Published:** 2023-12-21

**Authors:** John Bowblis, Christopher Brunt, Huiwen Xu, Robert Applebaum, David Grabowski

**Affiliations:** Miami University, Oxford, Ohio, United States; Georgia Southern University, Statesboro, Georgia, United States; University of Texas Medical Branch, Galveston, Texas, United States; Miami University, Oxford, Ohio, United States; Harvard Medical School, Boston, Massachusetts, United States

## Abstract

Nursing homes face rapidly increasing wages and change their input mix as they face workforce shortfalls. For 15,959 freestanding NHs from 2017-2021, the proportion of hours worked by agency staff and the median wage cost per hour of agency and directly employed nursing staff were calculated from Medicare cost reports and Payroll-Based Journal data. The median hourly wage cost of directly employed staff (wage and fringe) increased 18.1% for RNs (to $41.99 in 2021), 19.8% for LPNs ($32.29), and 27.5% for CNAs ($19.62). The median hourly wage cost of agency staff (amount paid to staffing agency) increased at a faster rate and was substantially higher: 22.8% for RNs ($64.19); 28.3% for LPNs ($52.42), and 34.9% for CNAs ($33.73). The use of agency staff increased, starting from approximately 2% of all nursing staff hours for each of the nursing staff types in Q1 2017 to 5.6% hours for RNs, 9.4% hours for LPNs, and 8.8% hours for CNAs in Q4 2021. These observations have implications for quality and access to nursing home care. High use of agency staff may affect quality of nursing home care, and substantial wage differences between agency and directly-employed staff may affect worker satisfaction and attachment of directly employed workers. Hourly wage costs for all nursing staff have increased more rapidly than nursing home revenues, which only increased 16.6% over the study period; this suggests that, without payment rate increases, nursing homes may reduce labor inputs to contain costs, or may go out of business.

